# Organic Nitrate Maintains Bone Marrow Blood Perfusion in Ovariectomized Female Rats: A Dynamic, Contrast-Enhanced Magnetic Resonance Imaging (MRI) Study

**DOI:** 10.3390/pharmaceutics5010023

**Published:** 2012-12-21

**Authors:** Yi-Xiang J. Wang, Chun Hay Ko, James F. Griffith, Min Deng, Hing Lok Wong, Tao Gu, Yu Huang

**Affiliations:** 1 Department of Imaging and Interventional Radiology, The Chinese University of Hong Kong, Shatin, Prince of Wales Hospital, Shatin, New Territories, Hong Kong SAR, China; E-mails: griffith@cuhk.edu.hk (J.F.G.); gwendolynminmin@gmail.com (M.D.); gutao001@sina.com (T.G.); 2 Institute of Chinese Medicine, The Chinese University of Hong Kong, New Territories, Hong Kong SAR, China; E-Mail: gohey@cuhk.edu.hk; 3 Jockey Club Centre for Osteoporosis Care and Control, School of Public Health and Primary Care, The Chinese University of Hong Kong, Prince of Wales Hospital, Shatin, New Territories, Hong Kong SAR, China; E-mail: vhlwong@yahoo.com; 4 Institute of Vascular Medicine & School of Biomedical Sciences, The Chinese University of Hong Kong, Shatin, New Territories, Hong Kong SAR, China; E-Mail: yu-huang@cuhk.edu.hk

**Keywords:** nitrates, osteoporosis, DCE MRI, bone mineral density, osteoblast, osteoclast

## Abstract

This study investigated the effects of nitrate on bone mineral density (BMD) and bone marrow perfusion in ovariectomized (OVX) female rats, and also the effects of nitrate on *in vitro* osteoblastic activity and osteoclastic differentiation of murine monocyte/macrophage RAW 264.7 cells. Female Sprague–Dawley rats were divided into OVX + nitrate group (isosorbide-5-mononitrate, ISM, 150 mg/kg/ day b.i.d), OVX + vehicle group, and control group. Lumbar spine CT bone densitometry and perfusion MRI were performed on the rats at baseline and week 8 post-OVX. The OVX rats’ BMD decreased by 22.5% ± 5.7% at week 8 (*p* < 0.001); while the OVX + ISM rats’ BMD decreased by 13.1% ± 2.7% (*p* < 0.001). The BMD loss difference between the two groups of rats was significant (*p* = 0.018). The OVX rats’ lumbar vertebral perfusion MRI maximum enhancement (*E*max) decreased by 10.3% ± 5.0% at week 8 (*p* < 0.005), while in OVX + ISM rats, the *Emax* increased by 5.5% ± 6.9% (*p* > 0.05). The proliferation of osteoblast-like UMR-106 cells increased significantly with ISM treatment at 0.78 µM to 50 μM. Treatment of UMR-106 cells with ISM also stimulated the BrdU uptake. After the RAW 264.7 cells were co-treated with osteoclastogenesis inducer RANKL and 6.25 μM ~ 100 μM of ISM for 3 days, a trend of dose-dependent increase of osteoclast number was noted.

## 1. Introduction

The common pharmacologic agents to prevent osteoporosis include estrogen replacement therapy, bisphosphonates, and selective estrogen receptor modulators such as raloxifene [1,2]. Each medication has adverse effects, often resulting in discontinuation. Recent evidences suggest that nitrates, drugs typically prescribed for the treatment of angina, may be effective in preventing osteoporosis. Organic nitrates reduce the cardiac load and enhance the oxygen supply to the myocardial tissue by vasodilatation of coronary and peripheral blood vessels [3]. Animal studies demonstrate that nitric oxide donors, such as nitroglycerin, isosorbide mononitrate and isosorbide dinitrate, can prevent bone loss associated with estrogen deficiency and glucocorticoid administration, and in estrogen deficiency-induced bone loss this treatment can be as effective as estrogen therapy [4,5,6]. Clinical studies also indicate that older women taking nitrates for angina have higher bone mineral density (BMD) at the hip and heel compared to non-users and have a lower risk of fractures [7,8]. These evidences suggest that estrogenic beneficial effect on bone in post-menopausal women can be achieved simply by administrating a nitrate (instead of estrogen). Nitrates have several advantages over the medications currently used to prevent and treat osteoporosis. There have been no reports of an increased risk of cardiovascular disease or breast cancer with long-term use of nitrates [9]. Nitrates are generally more available worldwide, more convenient to take, and less expensive.

The mechanism of how nitrates influence bone mechanism, however, remains unclear. It was suggested that the nitric oxide (NO) donor, nitrates, both induce decreased bone resorption and increased bone formation. In ovariectomised women, nitroglycerin was reported to be as efficient as estrogen and seemed to suppress biomarkers of osteoclast-mediated resorption (urinary *N*-telopeptides) and increase osteoblast activity biomarkers (alkaline phosphatase and osteocalcin) [10]. One clinical trial indicates that otherwise healthy women randomised to 3 months of treatment with isosorbide mononitrate had a 36.3% decrease in *N*-telopeptide and a 15.9% increase in bone-specific alkaline phosphatase [11]. 

Bone loss induced by ovariectomy (OVX) in female rats has been validated as a clinically relevant model of postmenopausal bone loss [12]. It was reported that in addition to the decrease of BMD, there is also a decrease of bone marrow perfusion in OVX rats [13]. Its mechanism is not fully understood yet, but a reduction of erythropoetic marrow and an increase of marrow fat in OVX rats, as well as endothelial dysfunction may at least partially contribute to this bone marrow perfusion decrease [13]. This current study investigated the effects of isosorbide-5-mononitrate (ISM) on BMD and bone marrow perfusion in OVX rats. To date, there remains a lack of basic cellular studies related to the nitric oxide donor ISM in bone metabolism. In order to substantiate our findings in animal studies, cell studies were performed. ISM’s effects on *in vitro* osteoblastic activity as well as osteoclastic differentiation of murine monocyte/macrophage RAW 264.7 cells were assessed.

## 2. Results and Discussion

In group 1, one rat died due to the surgical procedures, one rat did not follow the ISM dosing regimen and was excluded, and one failed the MRI examination due to insufficient anesthesia. In group 2, two rats failed tail vein cannulation. Ten rats in group 1 and 11 rats in group 2 completed the scheduled CT and MRI studies. Histology examination was performed on 6 rats from each group.

**Figure 1:**
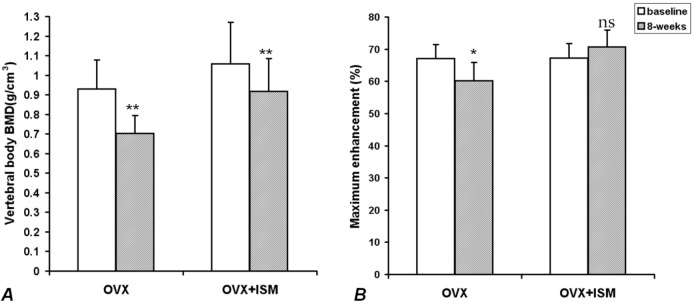
(**A**) Lumbar spine bone mineral density (BMD) results of ovariectomized (OVX) rats and OVX + isosorbide-5-mononitrate (ISM) rats at baseline and 8 weeks post surgery. There is less BMD loss in OVX + ISM rats than in OVX rats; (**B**) Dynamic contrast enhanced (DCE) magnetic resonance imaging (MRI) maximum enhancement results of OVX rats and OVX + ISM rats at baseline and 8 weeks post surgery.

In OVX rats, compared with the baseline values, lumbar spine vertebral BMD decreased by 22.5% ± 5.7% at week 8 (*p* < 0.001); while in OVX + ISM rats, lumbar spine vertebral BMD decreased by 13.1% ± 2.7% (*p* < 0.001) (Figure 1A). The difference in BMD loss between the two groups was significant (*p* = 0.018). In OVX rats, compared with the baseline values, *Emax* decreased by 10.3% ± 5.0% at week 8 (*p* < 0.005), while in OVX + ISM rats, *E*max increased by 5.5% ± 6.9% (*p* > 0.05) (Figure 1B). 

**Figure 2:**
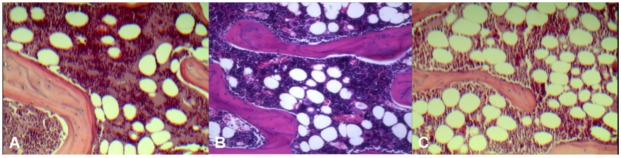
Representative microphotographs of lumbar vertebral marrow of control rat (**A**); OVX + ISM rat (**B**); and OVX rat (**C**). OVX rats have the least amount of erythropoetic marrow while control rats have the largest amount of erythropoetic marrow. The marrow composition of OVX + ISM rat lies between control rats and OVX rats.

Histology results (Figure 2) demonstrated control rats had the least amount of fatty marrow (36 ± 4 cells/×200 field of view, 21% ± 2.4% of microscopic view areas) and therefore largest amount of erythropoetic marrow. OVX rats had the largest amount of fatty marrow (64 ± 6 fat cells/×200 field of view, 42% ± 4.1% of microscopic view areas) and therefore least amount of erythropoetic marrow. The marrow composition of OVX + ISM rat lay between that of control rats and OVX rats (43 ± 6 cells/×200 field of view, 30% ± 6.0% of microscopic view areas). 

**Figure 3:**
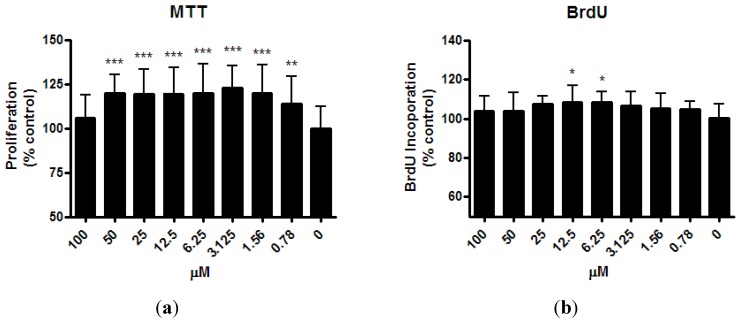
Effect of ISM on (**a**) cell proliferation and (**b**) bromodeoxyuridine (5-bromo-2'-deoxyuridine (BrdU) incorporation of osteoblast-like UMR-106 cells for 24 h at different concentrations. All results are expressed as mean ± SD of three independent experiments each in triplicates. Significant difference: ** *p* < 0.01; *** *p* < 0.001 for difference from respective baseline cultures without treatment.

MTT assay demonstrated the proliferation of osteoblast-like UMR-106 cells increased significantly after ISM treatment for 24 h at the minimum concentration of 0.78 µM and up to 50 μM (by 14.0% ~ 22.6%, Figure 3a). However, this proliferation enhancement effect was abolished at the highest concentration of 100 μM ISM. Treatment of UMR-106 cells with ISM for 24 h also stimulated the BrdU uptake (Figure 3b). BrdU incorporation increased significantly by 7.7% and 8.4% with ISM concentration at 6.25 and 12.5 μM, respectively (*p* < 0.01), compared with the control without treatment. However, at lower ISM concentrations of 0.79 μM ~ 3.125 μM and higher ISM concentrations of 25 μM ~ 100 μM, a trend of BrdU uptake increased was seen but statistical significance was not achieved, likely due to lower magnitude of changes and limited repetitive tests performed. 

**Figure 4:**
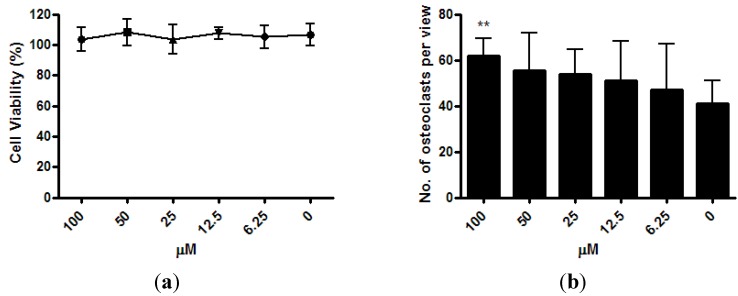
Osteoclastogenic properties of ISM in RAW264.7 cells upon receptor activator of nuclear factor kappa-B ligand (RANKL) induction. (**a**) Viability of osteoclast precursor RAW 264.7 cells is not affected by ISM; (**b**) A trend of dose-dependent increase of osteoclast number is noted. All results are expressed as mean ± SD of three independent experiments each in triplicates. Significant difference: ** *p* < 0.01 for difference from respective baseline cultures without treatment.

The viability of osteoclast precursor RAW 264.7 cells was not affected by ISM with concentrations up to 100 μM after 3 days of treatment (Figure 4a). After the RAW 264.7 cells were co-treated with osteoclastogenesis inducer RANKL and 6.25 μM ~100 μM of ISM for 3 days, a trend of dose-dependent increase of osteoclast number was noted. At 100 μM, the osteoclast number significantly increased by 51.0% ± 18.4% (*p* < 0.01) when compared with the control without treatment (Figure 4b). 

Nitric oxide (NO) has emerged as a potent regulator of bone metabolism mediating the effects of pro-inflammatory cytokines [14,15,16,17], estrogen [18,19], mechanical strain [20], fluid flow, and flow-induced shear stress [21,22]. It was shown that L-arginine-derived nitric oxide (NO) has a direct inhibitory effect on osteoclast-mediated bone resorption [23,24,25]. NO levels increase after estrogen therapy, and estrogenic effects on bone are mediated via NO [5]. NO metabolites decrease in postmenopausal and other amenorrhoeic women and are increased by estrogen replacement [26,27,28,29]. During the menstrual cycle, blood NO metabolite concentrations correlate with estrogen levels, and they are higher in the follicular than in the secretory phase [30]. Both estrogen and nitroglycerin significantly decrease the OVX-induced increase of urinary excretion of deoxypyridinolines, suggesting a decrease of osteoclastic activity. Nitroglycerin therapy may maintain higher levels of serum osteocalcin (increased osteoblastic activity) while reduce urinary deoxypyridinoline levels (decreased osteoclastic activity) [5].

It is known that rats between the age of 6 and 8 months old tend to maintain their lumbar spine BMD and marrow perfusion consistent [31]. The BMD results in this study shows orally administered ISM at 150 mg/kg per day b.i.d partially prevented bone loss due to OVX, and perfusion MRI shows ISM maintained the blood perfusion in lumbar vertebral marrow. Hukkanen *et al.* suggested nitroglycerin on bone metabolism is likely to include both a bone cell-mediated and vasoreactive responses [32]. However, the relative contributions of vascular and bone cell function as a response to nitroglycerin remains unclear [32]. Vertebral marrow composition assessment demonstrated that control rats had the most amount of erythropoetic marrow, OVX rats had the least amount of erythropoetic marrow, while the marrow of OVX + ISM rat lied between control rats and OVX rats. ISM, which is a vasodilator that relaxes and widens blood vessels, likely improves blood perfusion in bone marrow. The higher bone marrow perfusion in OVX + ISM rats than in OVX rats in this study is likely due to the combined effects of higher erythropoetic marrow components, which demand higher blood perfusion than fatty marrow, and relaxed vasculature tone in the OVX + ISM rats as opposed to the OVX rats. It is known that OVX induces vasoconstriction in rats [13].

We found ISM significantly increased the osteoblastic activities at the concentration range between 0.78 μM and 50 μM. However, the osteoblast proliferation enhancement effect was abolished at the concentration of 100 μM ISM. Interestingly, we found that at a high concentration of 100 μM ISM, a significant increase in osteoclastic effect was observed. This bi-phase response is likely mediated by the regulatory action from signaling NO molecules at different concentrations. ISM acts as a NO donor to the osteoblasts and osteoclasts, and changes their bone remodeling activities [33]. It is possible that at high concentrations NO favours osteoclast formation and trigger bone resorption activities; whereas at low concentrations NO favours the osteoblastic activities [34]. However, the high dose of 100 μM is difficult to achieve *in vivo* as evidenced by pharmacokinetic studies. Trenk *et al.* reported that single oral dose of 20 mg ISM increased the plasma concentration up to 406 ng/mL (2.12 μM) within one hour [35]. Similar result was also found in rat model after the administration of isosorbide dinitrate [36]. 

The dose used in this study is the same as the dose used by Reis *et al.* to treat cyclosporine A-induced hypertensive rats [37]. Similarly to Reis *et al.*’s study [37], in order to have a daily nitrate-low/free period of approximately 4–5 h to overcome organic nitrate tolerance, the twice daily administration of ISM was administered asymmetrically at 10:00 am and 4:00 pm. It has been reported that nitroglycerin administration can completely maintain or restore BMD loss due to OVX procedure [6,10]. However, in this study with the current dosing regimen, *i.e.*, ISM 150 mg/kg per day b.i.d, BMD was only partially maintained. It is known the dosing regimen plays an important role in osteoporosis prevention by nitrates. In an *in vivo* study it was shown that nitroglycerin improved BMD in rats when applied once daily yet the effects diminished when applied more frequently [10]. 

To prevent nitrate tolerance in ischemic heart disease, intermittent therapy with an adequate nitrate-free interval is proposed [38]. Continuous administration of nitroglycerin and other NO donating organic nitrates can lead to vascular and haemodynamic tolerance [39]. In a prospective population study, elderly women who daily used nitroglycerin, isosorbide monodinitrate, or isosorbide dinitrate, had slightly greater hip BMD but no difference in heel BMD in comparison to nonusers of nitrates. In contrast, women who used nitrate compounds only intermittently had substantially greater hip and heel BMD [7]. While the dosing regimen in this study proved the principle that ISM maintains bone mass as well as blood perfusion in bone marrow, the optimal dosing regimen, in terms of dosage and dosing timing, remains to be further explored. Other limitations of the current study include the fact that animal number used in this study is small; further studies are required to validate these findings. The effect of lower concentrations of ISM on osteoclast should be further investigated. Further in-depth mechanistic studies are also required to understand the osteoblastic and osteoclastic effects of ISM using different signaling inhibitors and immuno-precipitation, and how these results can be translated into *in vivo* scenarios. 

## 3. Experimental Section

### 3.1. Animal Studies

The animal experimental protocol was approved by the local University Animal Experiment Ethics Committee. Thirty two female Sprague–Dawley rats were used. All rats were bred at the Laboratory Animal Services Centre of our University, and were 7 months old at baseline. Animals were housed two to three animals per stainless steel cage at 22 °C temperature with a 12-h light and 12-h dark cycle and received a standard rat chow (Prolab RMH 2500, PMI Nutrition International LLC, Brentwood, MO, USA) and water *ad libitum*. For MRI examinations and surgery, the rats were anesthetized using a combination of xylazine (10 mg/kg) and ketamine (90 mg/kg).

**Figure 5:**
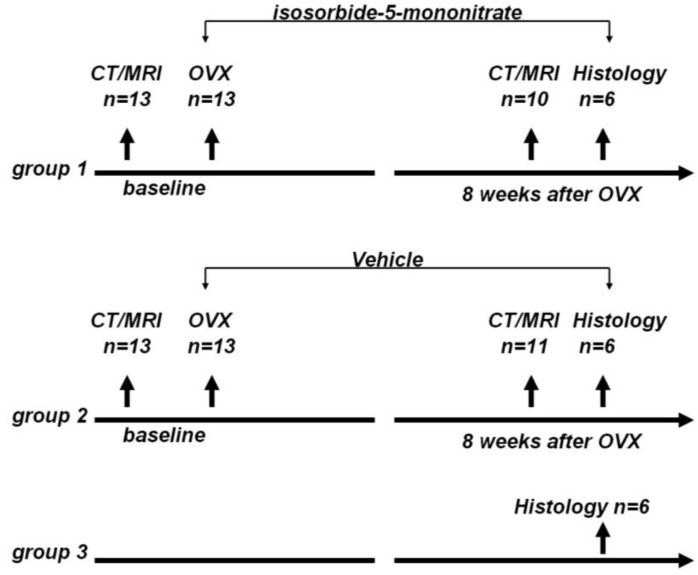
Animal study flow diagram. CT: Computed tomography, MRI: magnetic resonance imaging, OVX: ovariectomy.

The rats were randomly divided to three groups: group 1, OVX + nitrate treatment group (*n* = 13); group 2, OVX + vehicle treatment group (*n* = 13); and group 3, control group (*n* = 6). The animal study flow diagram is shown in Figure 5.

Computed tomography (CT) assessment of rat lumbar vertebral BMD was measured by using a clinical multidetector CT scanner (LightSpeed VCT 64; GE Healthcare, Milwaukee) with a continuous axial 0.625-mm section thickness. A more detailed description of the method has been reported elsewhere [40,41]. Lumbar vertebral BMD for each rat was defined as the mean BMD of vertebrae L1 through L6. 

Dynamic contrast enhanced (DCE) perfusion MRI study of the rat lumbar spine was performed according to the descriptions of Wang *et al.* [31,42,43]. In brief, MRI was performed on a 3 T clinical whole-body imaging system (Achieva, Philips Healthcare, Best, The Netherlands). Before MR imaging, rat tail vein was cannulated with a 24G heparinized catheter (Introcan Safety, B Braun Medical Inc, Bethlehem, PA, United States). For MRI of the spine, a custom-made quadrature volume RF coil of 7 cm internal diameter was used as signal transmitter and receiver. Rats were placed in the coil supine, and a central sagittal plane was prescribed. For all MRI examinations, T1 weighted anatomical images with a slice thickness of 2 mm were obtained prior to DCE scans (Figure 2A). DCE MRI series included: gradient echo sequence, TR = 5.4 msec, TE = 2.3 msec, flip angle = 12, slice thickness = 2 mm, acquisition resolution = 0.63 × 0.63 mm, temporal resolution = 0.6 sec/acquisition, average = 1. MRI contrast agent was Gd-DOTA (Guerbet Group, Roissy CDG cedex, France). A dose of 0.15 mmol/kg (0.075 mL for a 250 gram rat) was hand-injected after initial baseline 60 image acquisitions as quick bolus and followed by a flush of 0.5 mL normal saline. The DCE MRI scan duration was 8 min. 

**Figure 6:**
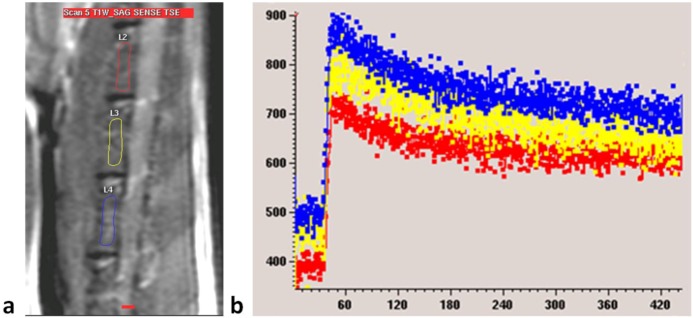
(**a**) MRI sagittal view of a lumbar spine of a rat. Three regions of interest (ROIs) are drawn over lumbar vertebral bodies; (**b**) Dynamic MRI enhancement curve (signal intensity in arbitrary units *vs.* time in seconds) of the same rat as in (**a**). Curves of different colors indicate different ROIs. Similar enhancement is seen among the three vertebral bodies.

Dynamic MRI images were processed on a radiological workstation (Viewforum, Philips Medical System, Best, The Netherlands). On anatomical sagittal images regions of interest (ROIs) were drawn over the cancellous part of the lumbar vertebrae, excluding the vertebral cortex, to generate a dynamic MRI enhancement curve (signal intensity in arbitrary units *vs.* time in seconds, Figure 2B). For each examination, three intervertebral bodies closest to the center of the receiver coil, mostly L2–L4, were selected (Figure 6). The investigator undertaking analyses of enhancement curve data was blinded to the animal grouping.

Maximum enhancement (*E*max) was used in this study representing blood perfusion. It was defined as the maximum percentage increase in signal intensity from baseline, and calculated from the signal enhancement curve of the dynamic MRI scan (Equation 1, [13,16]).

*E*_max_ = [(SI_max_ − SI_base_)/SI_base_] × 100 (1)

where SI_max_ denoted maximum signal intensity post-enhancement and SI_base_ denoted signal intensity pre-enhancement. Mean value of the three intervertebral bodies represented the *E*_max_ value of the rat.

After baseline MRI and CT assessment, group 1 and group 2 rats underwent bilateral ovariectomy. For ovariectomy, fur on both sides of the body was shaved from a proximal thigh to the lower chest. Under anesthesia, bilateral ovariectomy was performed through flank incisions 1.5 cm inferior to the costal margin. Ovaries together with surrounding fat tissue were removed. The incision was closed using muscle and skin sutures. Success of ovariectomy was confirmed at necropsy by absence of ovarian tissue and atrophy of uterine horns at the end of the study. 

After ovariectomy, group 1 rats were dosed isosorbide-5-mononitrate (Is-5-Mn, ISM, Sigma). ISM was dosed orally 150 mg/kg per day through an oesophageal cannula, b.i.d (10:00 am and 4:00 pm) till the end of the study [37]. Group 2 rats were dosed with saline. At 8 weeks post surgery, CT densitometry and perfusion MRI were repeated on both group 1 rats and group 2 rats. ISM was also administered on the day of CT and MRI examinations. Group 3 rats did not receive any intervention during the course.

At the end of *in vivo* study, for histologic examination, lumbar spines were excised and fixed in 10% buffered formalin for 3 days and then decalcified with 10% formic acid for 4 weeks. Decalcified samples were embedded in paraffin and cut into 6-mm-thick axial slices. Sections were cut at 5 µm thick and were then stained with haematoxylin and eosin. For each rat, four vertebral slices from L2–L5 vertebrae were randomly selected for evaluation. Percentage area of marrow fat, and fat cell number per field (200× magnification) was measured in each section by using an image processing software (Image-Pro Plus, version 5.1; Media Cybernetics, Acton, MA, USA). 

### 3.2. *In vitro* Studies

Osteoblast-like UMR-106 cells and murine monocyte/macrophage RAW 264.7 cells were purchased from the American Type Culture Collection (ATCC, Manassas, VA, USA) and subcultured to confluence in Dulbecco’s modified Eagle’s medium (DMEM; Life Technologies, Carlsbad, CA, USA) containing 10% fetal bovine serum (Life Technologies, Carlsbad, CA, USA), penicillin (100 U/mL; Life Technologies, Carlsbad, CA, USA), and streptomycin (100 μg/mL; Life Technologies, Carlsbad, CA, USA) in a humidified 5% CO_2_ atmosphere at 37 °C. 

Cell proliferation/viability was determined by the 3-[4,5-dimethylthiazol-2-yl]-2,5-diphenyltetrazolium bromide (MTT; Sigma, St. Louis, MO, USA) assay after 24 h of treatment with ISM at various concentrations in 96-well plates (UMR-106, 5 × 10^3^/well; and RAW 264.7, 5 × 10^2^ cells/well). The relative amount of viable cells was determined by measuring the reduction of MTT dye in live cells to blue formazan crystals at optical density at 540 nm and expressed as the percentage of control samples without treatment. To determine whether ISM could stimulate osteoblastic cell proliferation, its effect on BrdU incorporation for DNA synthesis was also determined by bromodeoxyuridine (5-bromo-2'-deoxyuridine, BrdU) incorporation assay kit (Roche, Mannheim, Germany) after 24 h of treatment according to manufacturer’s instructions. 

To determine the effects of ISM on RANKL-induced osteoclastogenesis of RAW 264.7 cells, tartrate-resistant acid phosphatase (TRAP)-positive cells were stained and counted after 3 days of treatment. In brief, RAW 264.7 cells were seeded in 96-well plates at a density of 5 × 10^2^ cells/well in DMEM. After 1 day of incubation, the differentiation of osteoclasts from RAW 264.7 cells was induced with 50 ng/mL of recombinant mouse soluble receptor activator of nuclear factor kappa-B ligand (RANKL; Sigma, Saint Louis, MO, USA) in α-minimal essential medium (Life Technologies, Carlsbad, CA, USA) with 10% FBS for 3 days. After treatment with RANKL and ISM, the RAW 264.7 cell differentiated osteoclasts were fixed and stained for TRAP, an osteoclast enzyme marker, by using an acid phosphatase kit (Sigma, Saint Louis, MO, USA) according to the manufacturer's instructions. TRAP-positive multinucleated cells showing more than three nuclei were counted as osteoclasts. Photomicrographs were taken with an inverted microscope at 40× magnification. The number of osteoclasts per field was calculated by averaging the counting from eight separated views.

Data were expressed as mean ± standard deviation. For comparison, Wilcoxon-signed rank test or Mann Whitney U test was used. All statistical analyses were performed using SPSS 14.0 (SPSS, Inc, Chicago, IL, USA). All statistical tests were two-sided. A *p* value of <0.05 was considered statistically significant.

## 4. Conclusions

The current study further confirms that ISM at selected concentrations have beneficial effects on bone mass. Perfusion MRI results seem to suggest ISM affects bone mass partially through its effect on maintaining blood perfusion to the bone. This study further demonstrates that perfusion MRI-derived readout can support the understanding of mechanism of pharmaceutics treatment [44]. Future works should include pharmacodynamic and pharmacokinetic studies to determine differential effects of nitrates by dose, frequency and duration of action. 
